# Efficacy of cervical physical therapy on pain sensitivity in patients with migraine compared to no treatment, sham treatment or usual medical care: a systematic review and meta-analysis

**DOI:** 10.1080/10669817.2026.2614009

**Published:** 2026-01-18

**Authors:** Andreas L. Amons, René F. Castien, Willem De Hertogh, George L. Burchell, Linda J. Schoonmade, Johannes C. van der Wouden, Joost Dekker, Henriëtte E. van der Horst

**Affiliations:** a Department of General Practice, Amsterdam University Medical Centers, Amsterdam, The Netherlands; b Amsterdam Public Health Research Institute, Amsterdam, The Netherlands; c Faculty of Behavioural and Movement Sciences, Vrije Universiteit Amsterdam, Amsterdam, The Netherlands; d Department of Rehabilitation Sciences and Physiotherapy, Faculty of Medicine and Health Sciences, University of Antwerp, Wilrijk, Belgium; e Medical Library, Vrije Universiteit Amsterdam, Amsterdam, The Netherlands; f Department of Rehabilitation Medicine and Department of Psychiatry, Amsterdam University Medical Centers, Amsterdam, The Netherlands

**Keywords:** Migraine, pressure pain threshold, physical therapy, cervical spine, review, meta-analysis

## Abstract

**Objective:**

To examine the efficacy of cervical physical therapy (e.g. cervical mobilization, exercise, and myofascial treatments) on pressure pain thresholds (PPTs) in patients with migraine, compared to no treatment, sham treatment, or usual medical care.

**Method:**

A systematic search was performed in PubMed, Embase.com, Web of Science, SPORTDiscus, Cinahl Plus and PEDro. Two independent reviewers assessed the selected randomized controlled trials (RCTs). The risk of bias was assessed using the Cochrane risk of bias tool 2, and the certainty of evidence was evaluated according to the GRADE approach. The meta-analysis included PPTs from cervical muscles and cephalic muscles.

**Results:**

Five papers out of 965 screened papers met our inclusion criteria, of which two originated from the same randomized controlled trial. Four RCTs had some risk of bias due to the absence of a pre-specified analysis plan and missing outcome data. One RCT had a high risk of bias due to a lack of information on concealment and blinding. All studies reported increased PPTs after cervical treatment. However, statistically significant differences were only found for the sternocleidomastoid muscle (mean difference (MD) 1.13 kg/cm^2^; 95% confidence interval (CI): 0.48 to 1.78) and the frontal muscle (MD 0.53 kg/cm^2^; 95% CI: 0.07 to 1.00). The certainty of evidence was low to very low.

**Conclusion:**

The efficacy of cervical physical therapy on PPTs is uncertain. More studies of high methodological quality are needed to elucidate the pain-modulatory effects of cervical physical therapy in migraine.

## 1. Introduction

In the last decade, there has been an increasing interest in non-pharmacological treatments for migraine [1–3]. Physical therapy (PT) is suggested as a potential adjunctive non-pharmacologic therapy [4]. However, in a cross-sectional study, only 19.5% of the migraine patients received PT, with 90% of the non-users having an interest in PT, expecting it to reduce migraine symptoms [5]. The high prevalence of neck pain (up to 75%) in migraine sufferers may explain this tendency to seek out PT treatments [6]. A survey among migraine patients showed that the majority who received PT were treated with manual therapy, including soft tissue treatments targeting the cervical spine [7]. Of those who received physical therapy, 54% reported perceived improvements in migraine frequency, intensity, and duration [7]. Evidence for the effectiveness of physical therapies targeting the cervical spine in people with migraine is scarce. Some systematic reviews report no effects after treatment [4,8], others report a decrease in migraine symptoms and disability levels along with improved quality of life [9–13]. How and why cervical PT might lead to a decrease in migraine attacks or duration is not clear. Understanding the mechanism of action of cervical PT on migraine may enhance the treatment’s effectiveness. The effectiveness of non-cervical PTs, such as aerobic exercise and relaxation, shows effects in reducing migraine frequency and disability [14,15]. A study by Kroll et al. (2018) assessed the modulatory effects of aerobic exercise in migraine patients and found no significant changes in pressure pain thresholds (PPTs), pericranial tenderness, and sensitivity to electrical stimulation following intervention [16].

In migraine, sensitization of the trigeminocervical complex (TCC) is recognized as part of the pathophysiological mechanism [17,18]. Nociception from peripheral vascular and cervical myofascial structures related to the TCC may increase sensitization of the TCC and, therefore, contribute to migraine [18].

Migraine is associated with neck pain and cervical musculoskeletal impairments, including decreased mobility and cervical muscle function, as well as increased cervical mechano-sensitivity [6,19]. Comorbid neck pain in migraine patients has been associated with a more severe clinical presentation and increased disability, including an increased risk of chronicity [20–22].

The increased pericranial tenderness in migraine patients with comorbid neck pain suggests that neck pain may act as a generator of migraine [23]. Although self-reported neck disability is not associated with cervical dysfunction, it is correlated with lower PPTs, higher allodynia scores, and greater self-reported migraine disability [24,25]. While the contributing role of neck pain and cervical disfunctions in migraine remains a topic of debate, assessment of cervical disfunctions and cervical mechano-sensitivity is recommended to explore potential adjunctive physical treatment options in people with migraine and comorbid neck pain [26].

Quantitative sensory testing (QST) can be used to assess sensitization. Within this framework, pressure algometry is most frequently applied to assess mechano-sensitivity by determining the PPTs [27]. The measurement of PPTs in people with migraine has demonstrated excellent reliability [28].

The suggested mechanism by which cervical PT modalities (e.g. mobilization, manipulation, exercise, or soft tissue treatments) may decrease mechano-sensitivity in the cervical region is through the reduction of cervical nociceptive input to the TCC, thereby reducing the sensitization of this pathway [29,30]. Till now, it is not known if, and how, cervical PT affects mechano-sensitivity in the cervical-cephalic and extra-cervical-cephalic regions in people with migraine. The modulatory effect of PT on PPTs has been reported in people with chronic musculoskeletal conditions [31]. However, outcomes vary across studied populations and depend on the applied modalities (i.e. spinal manipulation, exercises) used [32–34]. Summarizing the evidence of mechano-sensitivity after physical interventions is essential for developing plausible models of nociceptive modulation within the TCC, which may result in more specific and effective cervical physical treatments for migraine.

Therefore, this meta-analysis aims to examine the effect of cervical PT on PPTs in patients with migraine, compared to no treatment, sham treatment, or usual medical care.

## 2. Method

This review is reported following the PRISMA guidelines [35]. The protocol of this review was registered in PROSPERO (registration number: CRD42024534607).

### 2.1. Search strategy

A systematic search was performed in the databases: PubMed, Embase.com, Web of Science Core Collection, SPORTDiscus (via EbscoHost), Cinahl Plus (via EbscoHost) and PEDro. Keywords included controlled terms (e.g. MeSH in PubMed and Emtree in Embase), free text terms for (synonyms of) ‘physiotherapy’ combined with (synonyms of) ‘cervical spine’ combined with (synonyms of) ‘migraine.’ The search in the PEDro physiotherapy database was performed using the free text terms ‘migraine’ and ‘randomized controlled trials.’ A full overview of the search terms per database is attached in the supplementary information (see Appendix 1). The search was conducted by a medical librarian (GLB) from the inception of the databases until 10 October 2023, and updated in collaboration with a medical librarian (LS) on 11 July 2025. Duplicate articles were excluded by a medical information specialist (LS) using EndNote X21.5 (Clarivate^tm^). Additionally, the references from the included studies were searched for relevant publications. The terms ‘pressure pain thresholds’ or ‘pain sensitivity’ were not included in our search because these are often secondary outcomes and frequently not described in the title or abstract.

### 2.2. Eligibility criteria

Inclusion criteria were: randomized controlled trial, people with migraine headache (classified as migraine according to the ICHD-II or ICHD-III criteria) [36], cervical PT treatments (e.g. manual therapy, soft tissue intervention, cervical exercise, or dry-needling), application of pain threshold measurements (algometry), and involvement of human subjects (age older than 18 years). Inclusion criteria for the comparator or control group interventions were: waiting list, sham treatment, placebo, or usual medical care. Only publications in English were included. We excluded non-cervical PT treatments (e.g. electric stimulation therapies, aerobic exercise, hydrotherapy, or yoga).

### 2.3. Selection of publications

Two reviewers (AA and LW) independently screened the titles and abstracts using the Rayyan online research tool (www.rayyan.ai). After screening the title and abstract, full-text publications were retrieved and assessed for their eligibility. A third reviewer (WDH) was designated to resolve any disagreements between the reviewers.

### 2.4. Data extraction

Data were extracted by two independent reviewers (AA, LW) and included: title, authors, publication year, study design, recruitment, blinding and randomization procedures, population demographics (including age, gender, migraine frequency, and neck pain), method of PPT measurement, follow-up time points and PPT outcomes. If mean differences (MD) and standard deviation (SD) of PPT data were not presented in the publication, the authors were asked to provide the PPT data. Data recordings were documented with Review Manager 5.4 (revman.cochrane.org/info).

### 2.5. Risk of bias assessment

The Cochrane Risk of Bias (RoB) 2 tool for randomized controlled trials was used to assess the quality of the included studies [37]. Two reviewers (AA and TN) independently assessed the risk of bias. Judgments were made according to the RoB guidance tables [37]. Differences between the assessments were documented and discussed with a third reviewer (WDH) to reach a conclusion about the risk of bias.

### 2.6. Strategy for data synthesis

Data synthesis is presented in mean differences from baseline to post-intervention measurement; all PPT outcomes are expressed in kg/cm2. Changes in PPT measurements at the same muscles are reported in MD and 95% confidence intervals (CI). The effect size is expressed in standardized mean differences (SMD) with 95% CI. We considered an effect size of <0.4 as a small effect, 0.4 to 0.70 as a moderate effect and >0.7 as a large effect [38]. The PPT data were pooled if the population demographics and the method of measuring PPTs were described and found to be comparable. If raw data were provided, we calculated the means and standard deviations from the raw data. If raw data were unavailable, MDs and SDs were calculated according to the Cochrane Handbook [38]. Results were visually displayed by forest plots for each measurement site. A random effects model was used because of the expected heterogeneity of the population and treatments. Heterogeneity was tested with the I^2^ test. We interpreted the I^2^ statistic according to the Cochrane Handbook (0%-40%: not important; 30%-60%: moderate heterogeneity; 50%- 75%: substantial heterogeneity; 75% to 100%: considerable heterogeneity). Results were summarized in accordance with the GRADE recommendations.

### 2.7. Certainty of evidence (CoE)

The CoE was evaluated using the GRADE (Grading of Recommendations, Assessment, Development, and Evaluations) assessment tool [39]. According to the GRADE handbook, two reviewers systematically assessed the quality of evidence. The CoE was initially rated high for the RCTs and could be downgraded based on factors such as RoB, inconsistency of results, indirectness of evidence, imprecision and publication bias. The CoE per outcome is presented in a GRADE summary of findings table (Table 2).

## 3. Results

### 3.1. Search results

**Figure 1.**
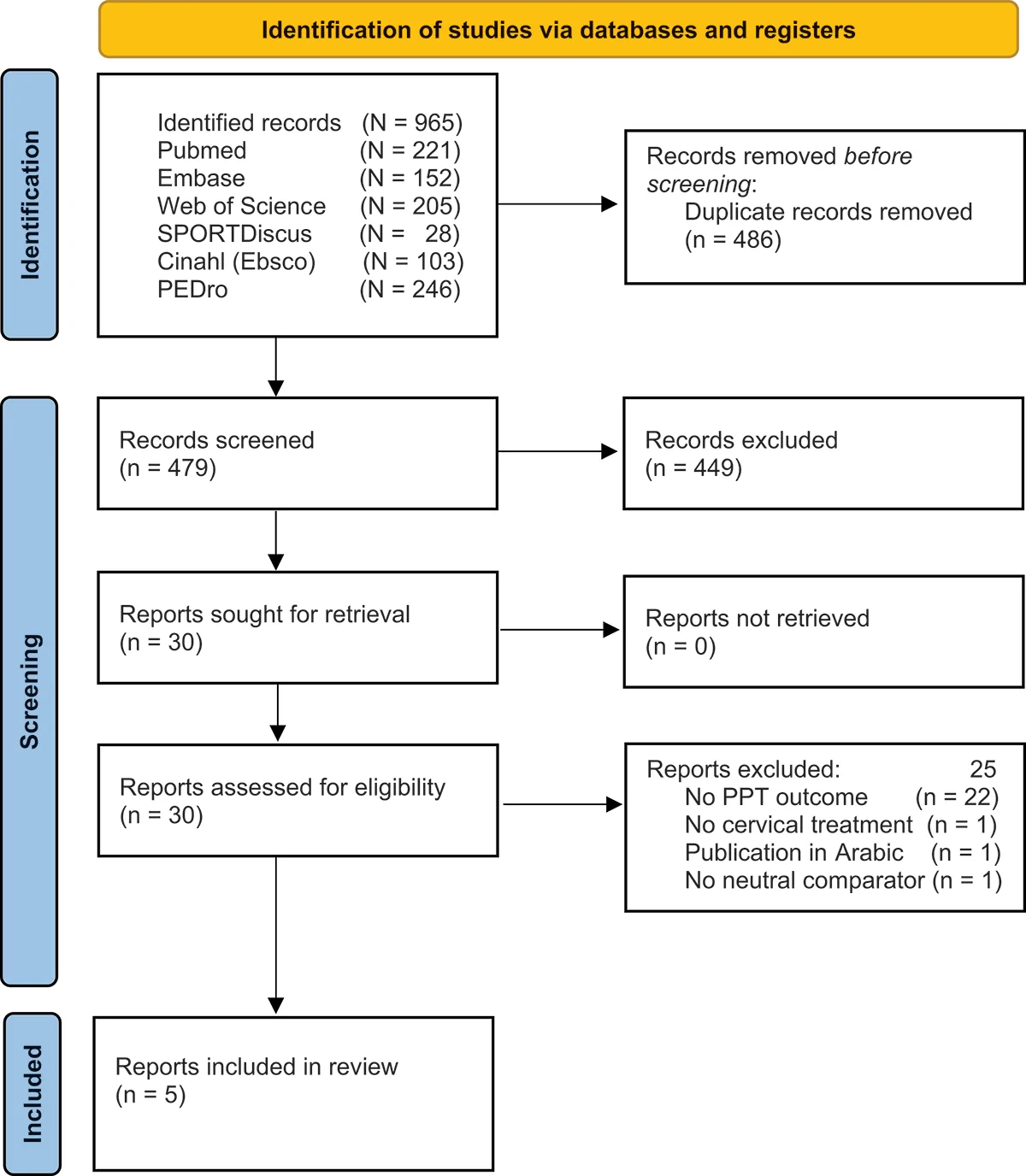
Flow chart of study inclusion.

The initial and updated search yielded 965 papers. After removing duplicates, 479 papers were screened for title and abstract. Subsequently, full-text screening was conducted on 30 papers, of which 5 met the inclusion criteria and were selected for this review. The flow chart (Figure 1) depicts a more comprehensive overview of this procedure.

Preferred reporting items for systematic reviews and meta-analyses (PRISMA) flow diagram.

### 3.2. Characteristics of studies

Five studies, comprising a total of 228 migraine patients, were included. In four studies, cervical PT consisted of myofascial treatment [40–43]. One of these four studies combined myofascial treatment with cervical mobilization and relaxation techniques [40], and the fifth study concerned neck strengthening exercises [44]. In two studies, cervical PT was combined with usual medical care [40,43]. The duration of the interventions varied across studies, ranging from one week of dry-needling [42], two weeks of myofascial treatment [41,43], and a four-week PT protocol [40], to eight weeks of neck strengthening exercises [44]. Two studies applied usual medical care as a control intervention [40,43], while the remaining three studies used placebo treatments as comparators [41,42,44]. Detailed information on the methods of the included studies is described in Table 1.

**Table 1.** Description of included studies.

Study	Participants	Intervention group	Control group	PPT Measurement	PPT outcome	Analysis/Results
Benatto (2022)	n = 42 (18-55y) (40 ♀ and 2 ♂) Migraine: ≧ 3d/month	n = 21 − 8 w CMSE protocol with specific exercises for Cx muscles (1/w for 20 min) - Home exercises: cervical stretches and exercises	n = 21 − 8 w sham ultrasound (1/w for 20 min) Home exercises	- Baseline, FU 8-w after treatment - Device: digital DDK-10 Kratos - Tested muscles: UT, SCM, Suboccipital, Temporal, Frontal and Levator scapulae	At each location, two PPT scores were averaged (kg/cm^2^). Left and right PPTs were combined. Anterior, medial and posterior temporal PPT scores were averaged into one mean score	- Dropout: IG (4/21), CG (1/21). - Analysis: linear mixed models; correction for baseline; dropouts excluded from analysis - The IG showed sign. increase in PPTs in the frontal muscle (*p* = < .05)
Bevilaqua- Grossi (2016)	n = 50 (18-55y) (50 ♀) Migraine ≧5d/month and Self-reported Cx pain ≧3 months	n = 25 − 4 w, 8 sessions (50 min) of PT and prescribed acute + prophylactic medication PT: relaxation, mobilization, traction, myofascial treatment.	n = 25 − 4 w prescribed acute + prophylactic medication	- Baseline, FU after treatment - Device: digital DDK-10 Kratos - Tested muscles: UT, SCM, suboccipital, temporal and frontal	At each location, two PPT scores were averaged (kg/cm^2^). Left and right PPTs were combined. Anterior, medial and posterior temporal PPT scores were averaged into one mean score	- Dropout: IG (6/25), CG (7/25) - Analysis: linear mixed models/correction for baseline. - The IG showed sign. increase in PPTs in the temporal muscle (*p* = < .05)
Ghanbari (2015)	n = 44 (mean age 37 y) (24♀ and 20♂) Migraine	n = 22 − 2 w, 5 sessions of PRT + Routine medication (e.g. NSAID, nortriptyline, propranolol and valproic acid)	n = 22 − 2 w Routine medication (e.g. NSAID, nortriptyline, propranolol and valproic acid)	- Baseline, FU at 1, 2 and 4 month - Device: analogue force gauge (Wagner-FDIX) - Tested muscles: UT, SCM, multifidus, rotatores and interspinal muscles of each segment	PPT scores from trigger points for all muscles (left and right) were averaged into one score (Kg/cm^2^)	- Dropout: not reported - Analysis: repeated measures test/no baseline correction - No sign. differences in PPTs were found between groups (*p* = 0.48)
Rezaeian (2019)	n = 46 (25-55y) (24 ♀ and 16 ♂) Migraine + Active trigger points occipital, SCM, UT	n = 23 (20 analyzed) − 2 w, 3 sessions/w for 20 min: soft tissue techniques on occipital, UT, SCM muscles	n = 23 (20 analyzed) − 2 w, 3 sessions of soft and superficial massage on Cx trigger points	- Baseline, FU immediate after treatment and at 1 month FU - Device: digital Lutron Force Gaug (FG-5005, RS-232) - Tested muscles: Occipital, UT, SCM	At each location, three PPT scores were averaged (kg/cm^2^). Left and right PPTs were combined	- Dropout: excluded from analysis; IG (3/23), CG (3/23). - Analysis: repeated measures test/no correction baseline - Sign. increase of PPTs post-treatment of IG vs CG at occipital, UT and SCM (*p* = 0.001)
Rezaeian (2020)	n = 46 (25-55y) (24 ♀ and 16 ♂) Migraine + active trigger points at SCM	n = 23 (20 analyzed) −1 w, 3 sessions with a 48 h interval of DN on SCM	n = 23 (20 analyzed) − 1 w, 3 sessions placebo DN	- Baseline, FU immediate after treatment and at 1 month FU - Device: digital Lutron Force Gaug (FG-5005, RS-232) - Tested muscles: SCM	At each location, three PPT scores were averaged (kg/cm^2^). Left and right PPTs were combined	- Dropout: excluded from analysis; IG (3/23), CG (3/23). Analysis: 3 × 2 repeated measures ANOVA/no baseline correction - Sign. increase of PPTs in DN IG vs CG at post-treatment (*p* = 0.006) and 1 month FU (*p* = 0.001)

CG = control group; CMSE: cervical muscles strength exercise; Cx = cervical; DN = dry needling; d/month = days per month; FU = follow-up; IG = intervention group; *n* = total of patients; min = minutes; NSAID = non-steroidal anti-inflammatory drug; PPT = pain pressure threshold; PRT = position release technique; PT = physical therapy; SCM = Sternocleidomastoid muscle; sign. = significant; UT = upper trapezius muscle; vs = versus; w = week; y = age in years; ≧ = at least; ♂ = male; ♀ = female.

### 3.3. Risk of bias

**Figure 2.**
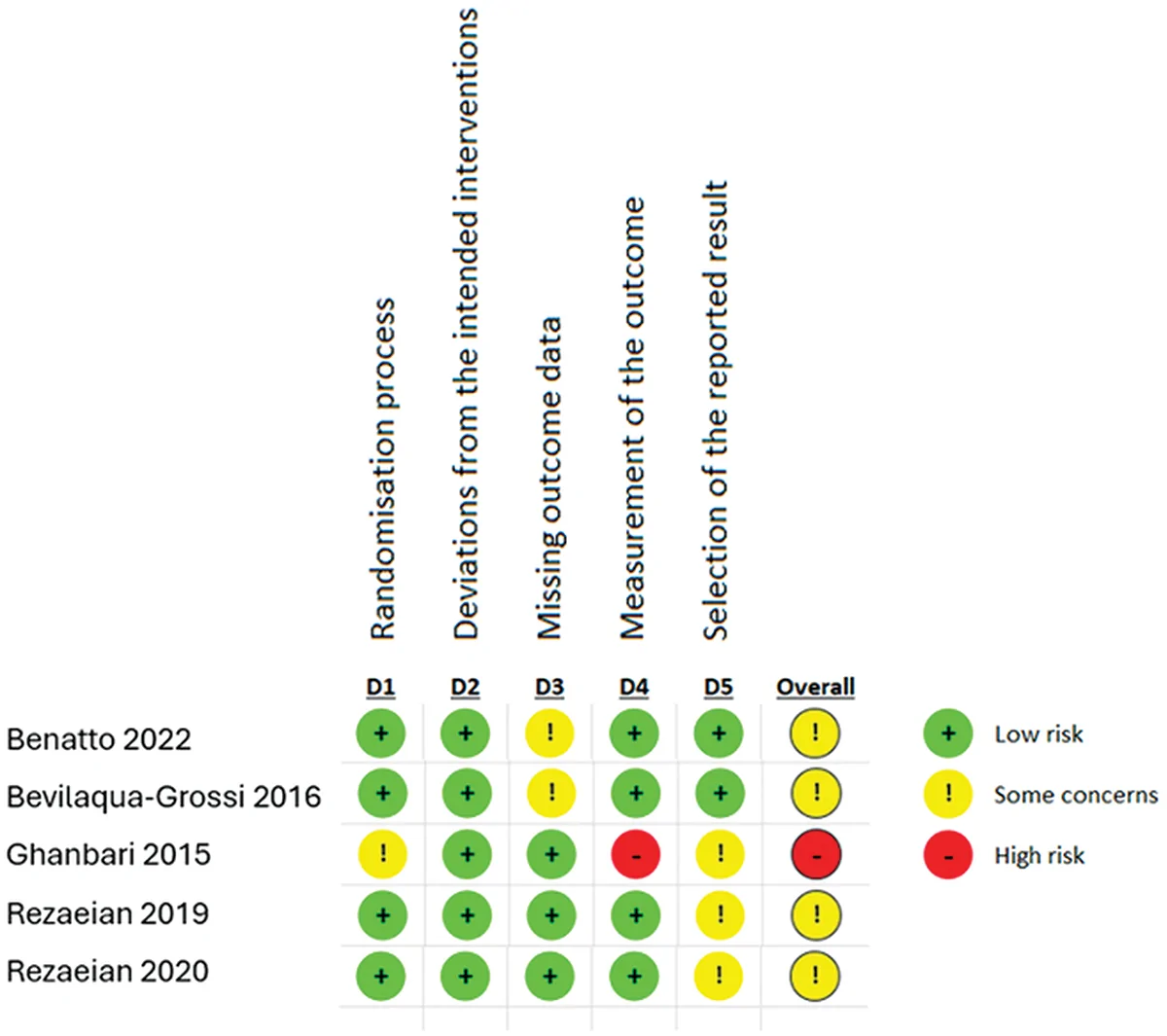
Risk of bias assessment.

In four out of five studies, the RoB was rated as ‘some concerns,’ and in one as a high RoB (Figure 2). The ‘some concerns’ rating was due to the absence of a pre-specified analysis plan [41–43], and missing outcome data due to drop-outs [40,44]. Information on concealment and blinding of assessors was lacking in one study, leading to a high RoB [43]. Disagreements concerning the risk of bias between reviewers were discussed with a third reviewer (WDH); the results are presented in Figure 2.

Risk of bias assessment of the included trials, using the Cochrane revised tool to assess risk of bias in randomized trials (RoB2).

### 3.4. PPT measurements

Four studies used a digital algometer (DDK-10 Kratos or Lutron FG5005) to measure PPTs [40–42,44], and Ghanbari et al. used an analogue algometer (Wagner FDX) [43]. All included studies averaged PPTs values of left and right muscles. The included studies did not assess PPTs outside of the cervical-cephalic area.

### 3.5. Synthesis of the results

The raw PPT data from Benatto et al. were used for the meta-analysis to calculate the means and SDs with paired sample T-tests [44]. Means and SDs from Rezaeian 2019 and 2020 were calculated according to the Cochrane Handbook using 95% confidence intervals, T-values and P-values [41,42]. Bevilaqua-Grossi et al. provided MDs and SDs for the meta-analysis [40]. The results reflect the short-term effects of cervical PT on PPTs, as long-term comparisons were not feasible; only two studies in our meta-analysis included follow-up PPT measurements [40,43].

#### 3.5.1. Results of PPTs on cervical muscles (Figure 3)

**Figure 3.**
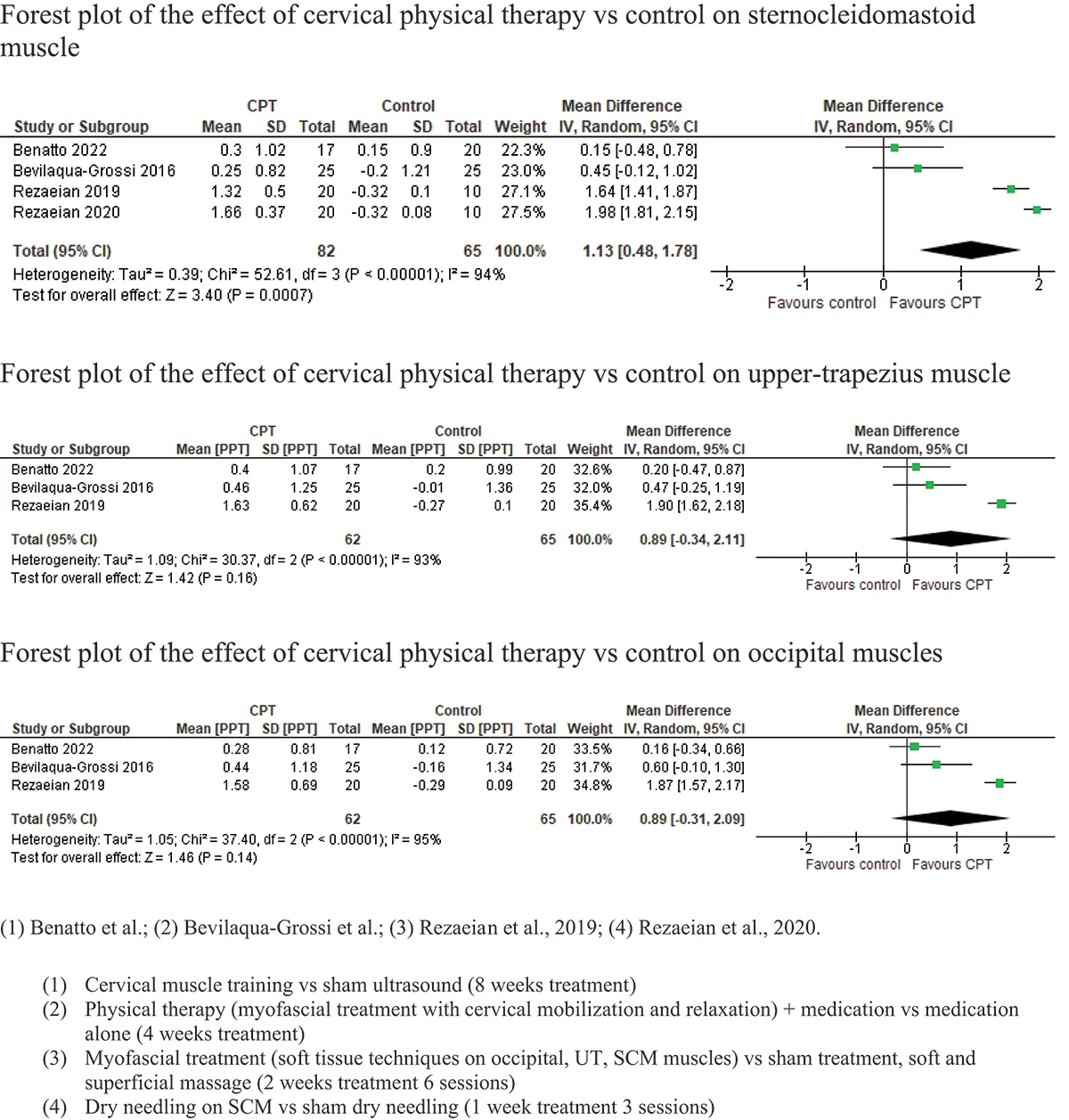
Forest plots on cervical PPTs on the sternocleidomastoid, upper trapezius and occipital muscles from baseline to post-intervention..

The meta-analysis evaluating the effect of cervical PT on PPT on the sternocleidomastoid muscle at post-treatment included four studies (*N* = 147). The analysis showed a significant improvement in PPT favoring cervical PT (MD 1.13; 95% CI from 0.48 to 1.78; I^2^ = 94%) with a large effect size (SMD 2.48; 95% CI 0.49–4.46). Rezaeian (2019, 2020) reported larger post-treatment improvements in PPTs at all cervical muscles than in other studies [41,42]. The publications by Rezaeian (2019) and Rezaeian (2020) originated from a three-armed study protocol. Both publications used the same participants as a control group. To prevent overweighing the results of these studies, the control group size was reduced by 50% to estimate the effect on the sternocleidomastoid muscle (Figure 3). The CoE was downgraded two points for non-overlapping 95% CIs and for considerable heterogeneity (I^2^ = 94%) and one point for imprecision caused by small sample sizes, with two out of four results representing both positive and negative effects. The CoE was rated as very low (see Table 2).

Forest plots on cervical PPTs on the occipital, upper trapezius and sternocleidomastoid muscles from baseline to post-intervention. CPT = cervical physical therapy; CI = confidence interval; SCM = sternocleidomastoid muscle; SD = standard deviation; UT = upper trapezius muscle; vs = versus.

**Table 2.** GRADE summary of findings table.

Intervention: cervical physical therapy (CPT) Comparison: control group			
Outcome	Participants	Certainty of evidence (GRADE)	Absolute effects mean difference (CI)
CPT vs control; PPT on SCM	n = 147 (4 RCTs)	**⊕◯◯◯** **Very low**^**a,b**^	**1.13** (0.48 to 1.78)
CPT vs control; PPT on UT	n = 127 (3 RCTs)	**⊕◯◯◯** **Very low**^**a,b**^	**0.89** (−0.34 to 2.11)
CPT vs control; PPT on Occipital muscles	n = 127 (3 RCTs)	**⊕◯◯◯** **Very low**^**a,b**^	**0.89** (−0.31 to 2.09)
CPT vs control; PPT on Frontal muscle	n = 87 (2 RCTs)	**⊕⊕◯◯** **Low**^**c**^	**0.53** (0.07 to 1)
CPT vs control; PPT on Temporal muscle	n = 87 (2 RCTs)	**⊕⊕◯◯** **Low**^**c**^	**0.51** (−0.02 to 1.05)

CI = confidence interval; MD = mean difference; PPT = pressure pain thresholds; RCT = randomized controlled trial; SD = standard deviation; SCM = sternocleidomastoid muscle; UT = upper trapezius muscle.

GRADE Working Group grades of evidence: Low certainty: Our confidence in the effect estimate is limited: The true effect may be substantially different from the estimate of the effect. Very Low certainty: We have very little confidence in the effect estimate: The true effect is likely to be substantially different from the estimated effect.

a. The 95%CIs of the studies do not overlap (some CIs are wide and others are small). The methodological heterogeneity (I^2^ test) is high. Conclusion: downgrading for inconsistency with two points.

b. The studies had small sample sizes. Two studies have wide CIs representing positive and negative effects. Conclusion: downgrading for imprecision by one point.

c. The two included studies had small sample sizes, and the confidence intervals of both studies are wide and include 0 as an effect. The number of events is small. Conclusion: due to imprecision, the level of evidence was downgraded by two points.

Data from three studies (*N* = 127) were used for the meta-analysis of post-treatment differences in PPTs for the upper trapezius and occipital muscles. For the upper trapezius, no significant effect was found (MD 0.89; 95% CI from −0.34 to 2.11; I^2^ = 93%; SMD 1.50; 95% CI − 0.38–2.29). Similarly, no significant effect was observed for the occipital muscles (MD 0.89; 95% CI from −0.31 to 2.09; I^2^ = 95%; SMD 1.40; 95% CI − 0.31–3.12). The CoE was graded as very low. The CoE was downgraded by two points for inconsistent results with differences in 95% CIs and considerable heterogeneity (I^2^ = 94%); imprecision caused by small sample sizes, with two out of four results representing positive and negative effects, also contributed to the downgrading by one point (see Table 2).

#### 3.5.2. Results of PPTs on cephalic muscles (Figure 4)

Two studies assessed the PPTs in muscles in the cephalic region, the temporal and frontal muscles (*N* = 87). Meta-analysis showed a significant increase in PPTs compared to control for the frontal muscle (MD 0.53; 95% CI from 0.07 to 1.00; I^2^ = 0%; SMD 0.48; 95% CI 0.06–0.91), and a non-significant effect in PPTs for the temporal muscle (MD 0.51; 95%CI from −0.02 to 1.05; I^2^ = 0%; SMD 0.42; 95% CI 0.00–0.85). Despite the low heterogeneity, the CoE was downgraded for imprecision by two points because of the small sample size and wide 95% CIs in both studies (Table 2).

**Figure 4.**
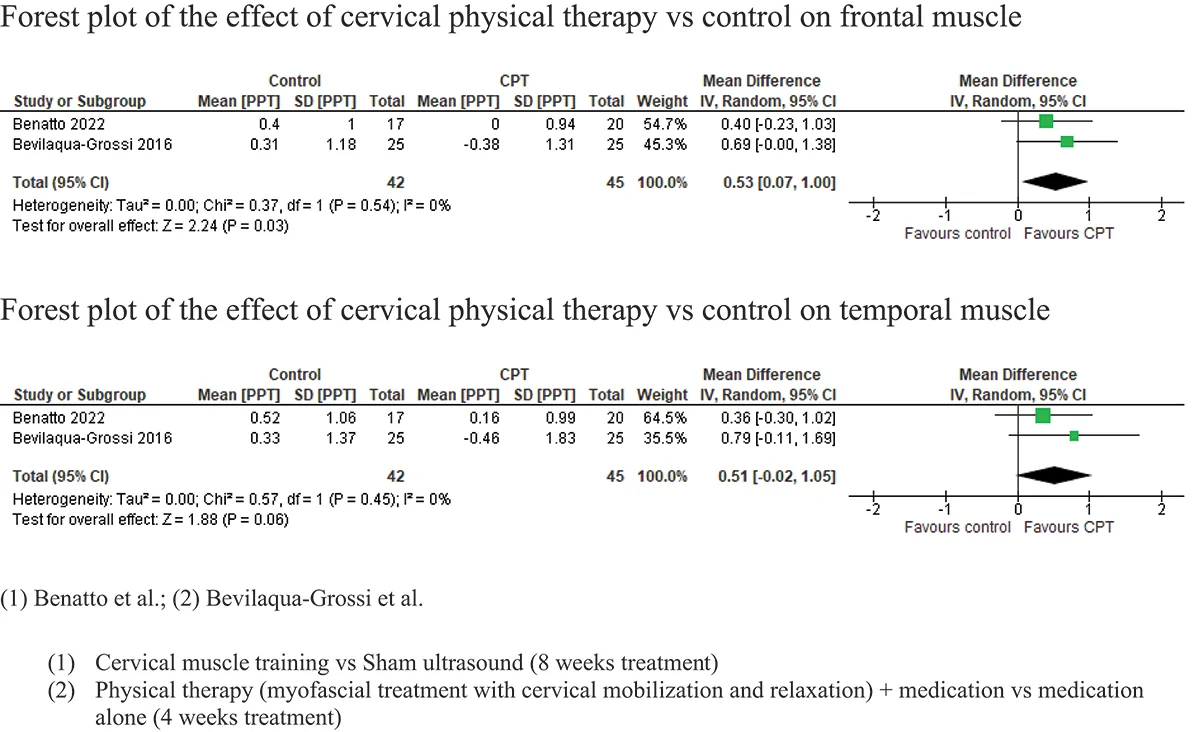
Forest plots cephalic PPTs on frontal and temporal muscles from baseline to post-intervention.

CPT = cervical physical therapy; CI = confidence interval; SD = standard deviation; vs = versus.

#### 3.5.3. Additional results of PPTs on cervical muscles

Ghanbari et al. reported an average increase in PPTs at trigger points across all tested cervical muscles (within the treatment group, *p* =  < 0.0001) [43]. However, the differences between the treatment and control groups were not statistically significant immediately after intervention nor at 1-, 2- and 4-month follow-up (*p* = 0.48). As Ghanbari et al. averaged PPT values across muscles, these data could not be included in the data pooling. Finally, Benatto et al. measured PPTs at the scapular levator muscle, reporting no significant difference between the treatment and control groups (*p* = 0.66) [44].

## 4. Discussion

### 4.1. Main findings

In this meta-analysis, we evaluated the effect of cervical PT on PPTs in patients with migraine. We found that cervical PT significantly increased PPTs at the sternocleidomastoid muscle (MD 1.13 kg/cm^2^; 95% CI: 0.48 to 1.78) and frontal muscle (MD 0.53 kg/cm^2^; 95% CI: 0.07 to 1.00), while PPT’s in other tested muscles (upper trapezius, temporal and occipital muscles) did not change significantly. The certainty of evidence is very low for the findings in cervical muscles and low for the findings in cephalic muscles.

The included papers in our review reported PPTs in cervical and cephalic regions, and thereby, our results are limited to the effects of cervical PT on mechano-sensitivity in the cervical-cephalic region. This prevents conclusions about central pain modulatory effects. The two studies by Rezaeian reported larger effects of cervical PT after treatment. These larger PPT changes can be explained by differences in migraine frequency and timing of PPT assessment. Participants in the Rezaeian trials had substantially lower headache frequency (3.5–3.85 days/month) compared with those in the studies by Benatto et al. and Bevilaqua-Grossi et al. (9.8 and 16 days/month). These differences in migraine frequency could have affected levels of sensitization and influenced the modulatory effects [23]. Additionally, Rezaeian assessed PPT immediately after the final treatment session, whereas other studies assessed PPT several days post-intervention [40] or did not specify the timing [44].

### 4.2. Comparison with existing evidence

Our findings on changes in PPTs after cervical PT are consistent with previous research. Similar results have been reported in studies that assessed two active cervical PT treatments in patients with migraine without a neutral control group, and therefore, were not included in our review [45–48]. Some studies reported no significant changes in PPTs after treatment [45,47], while others described increased PPTs after cervical PT treatment [46,48]. A recent study reported reductions in Central Sensitization Inventory (CSI) scores following cervical treatment compared with placebo or medication-only interventions, suggesting a potential pain-modulatory effect [49]. Results on PPTs after manual therapy have also been reported in recent reviews across various patient populations. Martínez-Pozas et al. (2023) found hypoalgesic effects in patients with chronic musculoskeletal pain [31], whereas Rodgers et al. (2024) reported no evidence of modulatory effects on PPTs following manual therapy in various musculoskeletal pain populations [34]. In tension-type headache, reductions in mechano-sensitivity after cervical PT treatments in cervical and cephalic muscles have been reported [50–54].

The clinical relevance of decreased PPTs in the sternocleidomastoid muscle and frontal muscles remains uncertain, as minimal clinically important differences in PPT values in these muscles have not been established for migraine. However, when compared with findings of altered mechano-sensitivity in individuals with neck pain after cervical PT, the changes observed in the sternocleidomastoid muscle (1.13 kg/cm^2^) exceeded the minimal detectable change in PPT values for neck pain (0.63 kg/cm^2^) [55].

### 4.3. Methodological considerations

Several methodological factors may have affected study outcomes. Differences in interventions and control conditions among the studies limited direct comparisons. For instance, cervical exercises, myofascial treatments, spinal manipulations and multimodal PT programs may have differing modulatory effects on mechano-sensitivity [31,32,56]. Additionally, treatment frequency and duration varied between studies, which could influence the outcomes [57]. Differences in PPT measurement protocols, such as averaging PPT measurements, muscle selection, and the type of algometer used, likely have contributed to the observed heterogeneity. Moreover, the timing of PPT measurements within the migraine cycle was not documented in any of the included studies. Since migraine is a cyclic condition with fluctuating sensitivity across phases, future research should apply measurements in the interictal phase [58,59].

The study of Bevilaqua-Grossi recruited participants with migraine and neck pain [40]. In this study, improvements in neck pain, due to treatment, could account for changes in PPT values, potentially confounding the results [22]. Two studies reported cephalic PPT measurements and applied identical measurement procedures and devices, resulting in a low I^2^ score (I^2^ = 0); however, the treatment duration and procedures differed [40,44].

The small number of studies found in our search, their limited sample sizes, considerable heterogeneity, and wide confidence intervals led to downgrading for heterogeneity and imprecision in the GRADE assessment, resulting in low or very low CoE.

### 4.4. Strengths and limitations

A strength of this meta-analysis is its adherence to PRISMA guidelines and GRADE recommendations, along with the comprehensive search conducted by two independent librarians [60]. We included only RCTs on cervical PT in migraine and synthesized the results in a meta-analysis where possible. However, there are several limitations. First, the pain-modulatory effects of cervical PT were only assessed through PPTs. Unfortunately, we have not retrieved studies on cervical PT that fulfill the inclusion criteria for this review, assessing quantitative sensory testing or temporal summation. Second, this review focused exclusively on PT treatments directed to the cervical spine, excluding general exercise interventions that may result in modulatory effects on pain sensitivity [14,33]. Lastly, the limited number of included studies represents a major limitation that restricts the ability to draw firm conclusions from our findings.

### 4.5. Implications for research and clinical practice

To enhance our understanding of the central pain-modulatory effects following cervical PT, we recommend standardizing the PPT sites (muscles), both within and outside the targeted treatment area. Furthermore, the PPT assessments should be applied in the interictal phase of the migraine cycle. Future sufficiently powered studies on the effectiveness of cervical PT interventions on PPTs that assess both local and widespread mechanosensitivity outcomes with longer follow-up assessments will provide a more comprehensive understanding of cervical PT’s working mechanism and its treatment potential for migraine patients.

Although we cannot draw conclusions about pain modulation by cervical PT in patients with migraine, in clinical practice, measuring mechanosensitivity in patients with migraine can still be recommended. PPTs can be easily assessed using an algometer. This simple, feasible, and reliable method allows the identification of migraine patients with increased pressure pain sensitivity [61]. Assessing PPTs may support clinical reasoning in the management of migraine and can be useful for monitoring changes over time or evaluating the modulatory effects of treatment.

## 5. Conclusion

The efficacy of cervical PT on PPTs is uncertain. More studies of high methodological quality are needed to elucidate the pain-modulatory effects of cervical PT in migraine.

## Supplementary Material

Appendix 2 PRISMA checklist review for submission JMMT.docx

Appendix 1 JMMT.docx

## Data Availability

The data used in this review were obtained from the included articles. Prof. D. Bevilaqua-Grossi provided additional information (means and SDs), and M.T. Benatto provided raw PPT data for this review.
