# Supplementary material for: Efficacy of cervical physical therapy on pain sensitivity in patients with migraine compared to no treatment, sham treatment or usual medical care: a systematic review and meta-analysis
Source: J Man Manip Ther. 2026 Jan 18;34(5):411–23. doi: 10.1080/10669817.2026.2614009 (PMC13600285; doi:10.1080/10669817.2026.2614009)
Supplement: Supplementary file 1 [file YJMT_A_2614009_SM2577.docx]

Appendix 1

**Supplementary Information**

**Supplementary information S1 – Search Histories and Results July 11, 2025**

## PubMed

| **Search** | **PubMed Query – July 11, 2025** | **Results** |
| --- | --- | --- |
| #1 | "Migraine Disorders"[Mesh] OR migrain*[tiab] | 51,842 |
| #2 | "Neck"[Mesh] OR "Cervical Cord"[Mesh] OR "cervical*"[tiab] OR "neck"[tiab] OR "upper spine"[tiab] OR "occipital"[tiab] OR "C1-C7"[tiab] OR "atlas"[tiab] OR "axis"[tiab] OR "nuchal"[tiab] OR "cervicogenic"[tiab] OR "suboccipital"[tiab] OR "sub-occipital"[tiab] OR "craniocervical"[tiab] OR "cranio-cervical"[tiab] OR "cervicocranial"[tiab] OR "cervical-cranial"[tiab] OR "cervicothoracic"[tiab] OR "cervico-thoracic"[tiab] | 927,460 |
| #3 | "Physical Therapy Modalities"[Mesh] OR "Resistance Training"[Mesh] OR "Musculoskeletal Manipulations"[Mesh] OR physiotherap*[tiab] OR "physical therap*"[tiab] OR "exercise*"[tiab] OR kinesitherapie[tiab] OR kinesitherap*[tiab] OR "manual therap*"[tiab] OR "movement therap*"[tiab] OR "mobilization*"[tiab] OR "mobilisation*"[tiab] OR (manipulati*[tiab] AND (orthopedic[tiab] OR spinal[tiab] OR spine[tiab] OR musculoskeletal*[tiab])) OR (neuromuscular[tiab] AND (reeducation*[tiab] OR re-education*[tiab])) OR (muscle*[tiab] AND (training*[tiab] OR stretching[tiab] OR strengthening[tiab] OR training*[tiab])) OR "resistance training*"[tiab] OR massag*[tiab] OR "myofascial therap*"[tiab] OR "myofascial release"[tiab] OR "dry needling"[tiab] OR "soft tissue therap*"[tiab] OR rehabilitation[tiab] | 863,645 |
| #4 | #1 AND #2 AND #3 | 461 |
| #5 | #4 NOT (("Adolescent"[Mesh] OR "Child"[Mesh] OR "Infant"[Mesh] OR "adolescen*"[tiab] OR "child*" [tiab] OR "schoolchild*"[tiab] OR "infant*"[tiab] OR "girl*"[tiab] OR "boy"[tiab] OR "boys"[tiab] OR "teen*"[tiab] OR "youth*"[tiab] OR "newborn"[tiab] OR "pediatr*"[tiab] OR "paediatr*"[tiab] OR "pube*"[tiab] OR "neonat*"[tiab]) NOT ("Adult"[Mesh] OR "adult*"[tiab] OR "man"[tiab] OR "men"[tiab] OR "woman"[tiab] OR "women"[tiab])) | 444 |
| #6 | #5 NOT ("Animals"[Mesh] NOT "Humans"[Mesh]) | 441 |
| #7 | #6 AND ("Clinical Trial" [Publication Type] OR "Clinical Trials as Topic"[Mesh] OR "random*"[tiab] OR "crossover*"[tiab] OR "cross over*"[tiab] OR "placebo*"[tiab] OR ("doubl*"[tiab] AND "blind*"[tiab]) OR ("singl*"[tiab] AND "blind*"[tiab]) OR "trial*"[tiab] OR "intervention"[tiab]) | 221 |

## Embase

| **Search** | **Embase.com Query – July 11, 2025** | **Results** |
| --- | --- | --- |
| #1 | 'migraine'/exp OR migrain*:ti,ab,kw | 100,385 |
| #2 | 'neck'/exp OR 'cervical spinal cord'/exp OR ('cervical*' OR 'neck' OR 'upper spine' OR 'occipital' OR 'C1-C7' OR 'atlas' OR 'axis' OR 'nuchal' OR 'cervicogenic' OR 'cervico-genic' OR 'suboccipital' OR 'sub-occipital' OR 'craniocervical' OR 'cranio-cervical' OR 'cervicocranial' OR 'cervico-cranial' OR 'cervicothoracic' OR 'cervico-thoracic'):ti,ab,kw | 1,274,884 |
| #3 | 'physiotherapy'/exp OR 'resistance training'/exp OR 'musculoskeletal manipulation'/exp OR 'kinesiotherapy'/exp OR 'soft tissue therapy'/exp OR 'dry needling'/exp OR 'myofascial release'/exp OR (physiotherap* OR 'physical therap*' OR exercis* OR kinesitherapie OR kinesitherap* OR 'manual therap*' OR 'movement therap*' OR 'mobilization*' OR 'mobilisation*' OR (manipulation* AND (orthopedic OR spinal OR spine OR musculoskeletal*)) OR (neuromuscular AND (reeducation* OR re-education*)) OR (muscle* AND (training* OR stretching OR strengthening OR training*)) OR 'strength training*' OR 'resistance training*' OR massag* OR 'myofascial therap*' OR 'myofascial release' OR 'dry needling' OR 'soft tissue therap*' OR rehabilitation):ti,ab,kw | 1,210,775 |
| #4 | #1 AND #2 AND #3 | 821 |
| #5 | #4 NOT (('juvenile'/exp OR 'adolescence'/exp OR ('adolescen*' OR 'child*' OR 'schoolchild*' OR 'infant*' OR 'girl*' OR 'boy' OR 'boys' OR 'teen*' OR 'youth*' OR 'newborn' OR 'pediatr*' OR 'paediatr*' OR 'pube*'):ti,ab,kw) NOT ('adult'/exp OR ('adult*' OR 'man' OR 'men' OR 'woman' OR 'women'):ti,ab,kw)) | 776 |
| #6 | #5 NOT ([animals]/lim NOT [humans]/lim) | 772 |
| #7 | #6 AND ('crossover procedure'/exp OR 'double blind procedure'/exp OR 'clinical trial'/exp OR 'randomized controlled trial'/exp OR 'single blind procedure'/exp OR 'intervention study'/exp OR ('random*' OR 'crossover*' OR 'factorial' OR ('cross' NEAR/2 'over*') OR 'placebo*' OR ('doubl*' NEAR/2 'blind*') OR ('singl*' NEAR/2 'blind*') OR 'trial' OR 'intervention'):ti,ab,kw) | 309 |
| #8 | #7 NOT ('clinical trial'/it OR 'conference abstract'/it) | 152 |

## Web of Science

| **Search** | **Web of Science Core Collection Query – July 11, 2025** | **Results** |
| --- | --- | --- |
| #1 | TS= (migrain*) | 66,572 |
| #2 | TS= ("cervical*" OR "neck" OR "upper spine" OR "occipital" OR "C1-C7" OR "atlas" OR "axis" OR "Nuchal" OR "cervicogenic" OR "cervico-genic" OR "suboccipital" OR "sub-occipital" OR "craniocervical" OR "cranio-cervical" OR "cervicocranial" OR "cervico-cranial" OR "cervicothoracic" OR "cervico-thoracic") | 1,317,991 |
| #3 | TS= (physiotherap* OR "physical therap*" OR exercis* OR kinesitherapie OR kinesitherap* OR "manual therap*" OR "movement therap*" OR "mobilization*" OR "mobilisation*" OR (manipulation* AND (orthopedic OR spinal OR spine OR musculoskeletal*)) OR (neuromuscular AND (reeducation* OR re-education*)) OR (muscle* AND (training* OR stretching OR strengthening OR training*)) OR "strength training*" OR "resistance training*" OR massag* OR "myofascial therap*" OR "myofascial release" OR "dry needling" OR "soft tissue therap*" OR rehabilitation) | 1,162,022 |
| #4 | #1 AND #2 AND #3 | 442 |
| #5 | #4 NOT TS=(("adolescen*" OR "child*" OR "schoolchild*" OR "infant*" OR "girl*" OR "boy" OR "boys" OR "teen*" OR "youth*" OR "newborn" OR "pediatr*" OR "paediatr*" OR "pube*" OR "neonat*") NOT ("adult*" OR "man" OR "men" OR "woman" OR "women")) | 413 |
| #6 | #5 AND TS=("random*" OR "crossover*" OR "cross over*" OR "placebo*" OR ("doubl*" AND "blind*") OR ("singl*" AND "blind*") OR "trial*" OR "intervention") | 215 |

## SPORTDiscus

| **Search** | **SPORTDiscus (Ebsco) Query** | **Results** |
| --- | --- | --- |
| S1 | DE "MIGRAINE" OR TI(migrain*) OR AB(migrain*) OR KW(migrain*) | 5,715 |
| S2 | DE ("NECK" OR "CERVICAL vertebrae") OR TI("cervical*" OR "neck" OR "upper spine" OR "occipital" OR "C1-C7" OR "atlas" OR "axis" OR "Nuchal" OR "cervicogenic" OR "cervico-genic" OR "suboccipital" OR "sub-occipital" OR "craniocervical" OR "cranio-cervical" OR "cervicocranial" OR "cervico-cranial" OR "cervicothoracic" OR "cervico-thoracic") OR AB("cervical*" OR "neck" OR "upper spine" OR "occipital" OR "C1-C7" OR "atlas" OR "axis" OR "Nuchal" OR "cervicogenic" OR "cervico-genic" OR "suboccipital" OR "sub-occipital" OR "craniocervical" OR "cranio-cervical" OR "cervicocranial" OR "cervico-cranial" OR "cervicothoracic" OR "cervico-thoracic") OR KW("cervical*" OR "neck" OR "upper spine" OR "occipital" OR "C1-C7" OR "atlas" OR "axis" OR "Nuchal" OR "cervicogenic" OR "cervico-genic" OR "suboccipital" OR "sub-occipital" OR "craniocervical" OR "cranio-cervical" OR "cervicocranial" OR "cervico-cranial" OR "cervicothoracic" OR "cervico-thoracic") | 25,580 |
| S3 | DE ("PHYSICAL therapy" OR "RESISTANCE training" OR "EXERCISE therapy" OR "MANIPULATION therapy" OR "STRENGTH training" OR "STRETCH (Physiology)" OR "MASSAGE therapy" OR "DRY needling") OR TI (physiotherap* OR "physical therap*" OR exercis* OR kinesitherapie OR kinesitherap* OR "manual therap*" OR "movement therap*" OR "mobilization*" OR "mobilisation*" OR (manipulation* AND (orthopedic OR spinal OR spine OR musculoskeletal*)) OR (neuromuscular AND (reeducation* OR re-education*)) OR (muscle* AND (training* OR stretching OR strengthening OR training*)) OR "strength training*" OR "resistance training*" OR massag* OR "myofascial therap*" OR "myofascial release" OR "dry needling" OR "soft tissue therap*" OR rehabilitation) OR AB (physiotherap* OR "physical therap*" OR exercis* OR kinesitherapie OR kinesitherap* OR "manual therap*" OR "movement therap*" OR "mobilization*" OR "mobilisation*" OR (manipulation* AND (orthopedic OR spinal OR spine OR musculoskeletal*)) OR (neuromuscular AND (reeducation* OR re-education*)) OR (muscle* AND (training* OR stretching OR strengthening OR training*)) OR "strength training*" OR "resistance training*" OR massag* OR "myofascial therap*" OR "myofascial release" OR "dry needling" OR "soft tissue therap*" OR rehabilitation) OR KW (physiotherap* OR "physical therap*" OR exercis* OR kinesitherapie OR kinesitherap* OR "manual therap*" OR "movement therap*" OR "mobilization*" OR "mobilisation*" OR (manipulation* AND (orthopedic OR spinal OR spine OR musculoskeletal*)) OR (neuromuscular AND (reeducation* OR re-education*)) OR (muscle* AND (training* OR stretching OR strengthening OR training*)) OR "strength training*" OR "resistance training*" OR massag* OR "myofascial therap*" OR "myofascial release" OR "dry needling" OR "soft tissue therap*" OR rehabilitation) | 311,484 |
| S4 | S1 AND S2 AND S3 | 83 |
| S5 | S4 NOT ((TI("adolescen*" OR "child*" OR "schoolchild*" OR "infant*" OR "girl*" OR "boy" OR "boys" OR "teen*" OR "youth*" OR "newborn" OR "pediatr*" OR "paediatr*" OR "pube*") OR AB("adolescen*" OR "child*" OR "schoolchild*" OR "infant*" OR "girl*" OR "boy" OR "boys" OR "teen*" OR "youth*" OR "newborn" OR "pediatr*" OR "paediatr*" OR "pube*") OR KW("adolescen*" OR "child*" OR "schoolchild*" OR "infant*" OR "girl*" OR "boy" OR "boys" OR "teen*" OR "youth*" OR "newborn" OR "pediatr*" OR "paediatr*" OR "pube*")) NOT (TI("adult*" OR "man" OR "men" OR "woman" OR "women") OR AB("adult*" OR "man" OR "men" OR "woman" OR "women") OR KW("adult*" OR "man" OR "men" OR "woman" OR "women"))) | 77 |
| S6 | S5 AND ( TI("random*" OR "crossover*" OR "factorial" OR ("cross" N2 "over*") OR "placebo*" OR ("doubl*" N2 "blind*") OR ("singl*" N2 "blind*") OR "trial" OR "intervention") OR AB("random*" OR "crossover*" OR "factorial" OR ("cross" N2 "over*") OR "placebo*" OR ("doubl*" N2 "blind*") OR ("singl*" N2 "blind*") OR "trial" OR "intervention") OR KW("random*" OR "crossover*" OR "factorial" OR ("cross" N2 "over*") OR "placebo*" OR ("doubl*" N2 "blind*") OR ("singl*" N2 "blind*") OR "trial" OR "intervention")) | 28 |

## Cinahl

| **Search** | **Cinahl (Ebsco) Query – July 11, 2025** | **Results** |
| --- | --- | --- |
| S1 | MH "Migraine" OR XB(migrain*) | 21,612 |
| S2 | MH ("Neck+" OR "Cervical Cord") OR XB("cervical*" OR "neck" OR "upper spine" OR "occipital" OR "C1-C7" OR "atlas" OR "axis" OR "Nuchal" OR "cervicogenic" OR "cervico-genic" OR "suboccipital" OR "sub-occipital" OR "craniocervical" OR "cranio-cervical" OR "cervicocranial" OR "cervico-cranial" OR "cervicothoracic" OR "cervico-thoracic") | 165,908 |
| S3 | MH ("Physical Therapy" OR "Kinesiotherapy" OR "Manual Therapy+" OR "Massage+" OR "Myofascial Release" OR "Therapeutic Exercise" OR "Muscle Strengthening+" OR "Exercise" OR "Stretching" OR "Joint Mobilization" OR "Dry Needling") OR XB (physiotherap* OR "physical therap*" OR exercis* OR kinesitherapie OR kinesitherap* OR "manual therap*" OR "movement therap*" OR "mobilization*" OR "mobilisation*" OR (manipulation* AND (orthopedic OR spinal OR spine OR musculoskeletal*)) OR (neuromuscular AND (reeducation* OR re-education*)) OR (muscle* AND (training* OR stretching OR strengthening OR training*)) OR "strength training*" OR "resistance training*" OR massag* OR "myofascial therap*" OR "myofascial release" OR "dry needling" OR "soft tissue therap*" OR rehabilitation) | 416,577 |
| S4 | S1 AND S2 AND S3 | 195 |
| S5 | S4 NOT ((MH ("Adolescence" OR "Child+") OR XB (adolescen* OR child* OR schoolchild* OR infant* OR girl* OR boy* OR teen OR teens OR teenager* OR youth* OR pediatr* OR paediatr* OR puber*)) NOT (MH ("Adult+") OR XB (adult* OR man OR men OR woman OR women))) | 187 |
| S6 | S5 AND ((MH "Experimental Studies+" OR "Clinical Trials") OR XB ("random*" OR "crossover*" OR "factorial" OR ("cross" N2 "over*") OR "placebo*" OR ("doubl*" N2 "blind*") OR ("singl*" N2 "blind*") OR "trial" OR intervention*)) | 103 |

## PEDro (0) oktober 10, 2023

| Search | Query | Results |
| --- | --- | --- |
| #5 | **After deduplication 0** | 0 |
| #4 | **3 studies included** | 3 |
| #3 | **21 studies full-text assessment** | 21 |
| #2 | **Exclusion on title and abstract** | 225 |
| #1 | **“migraine” AND “randomized controlled trials”** | 246 |
